# Supplementary material for: Proteomic Analysis of the Resistance Mechanisms in Sugarcane during Sporisorium scitamineum Infection
Source: Int J Mol Sci. 2019 Jan 29;20(3):569. doi: 10.3390/ijms20030569 (PMC6387155; doi:10.3390/ijms20030569)
Supplement: Supplementary file 1 [file ijms-20-00569-s001.pdf]

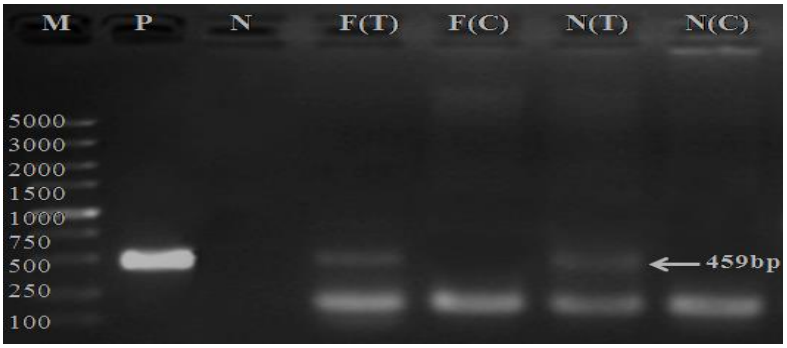


**Figure S1.** PCR amplification products of DNA isolated from control and smut inoculated sugarcane plantlets after 30 days, and *S. scitamineum* bE mating-type gene fragment is amplified at 459 bp. M, Molecular size marker (100 bp-5000 bp); P, positive control (*S. scitamineum*); N, negative control (Sterile water); F134; F(T) treatment and F(C) control; NCo310; N(T) treatment and N(C) control.

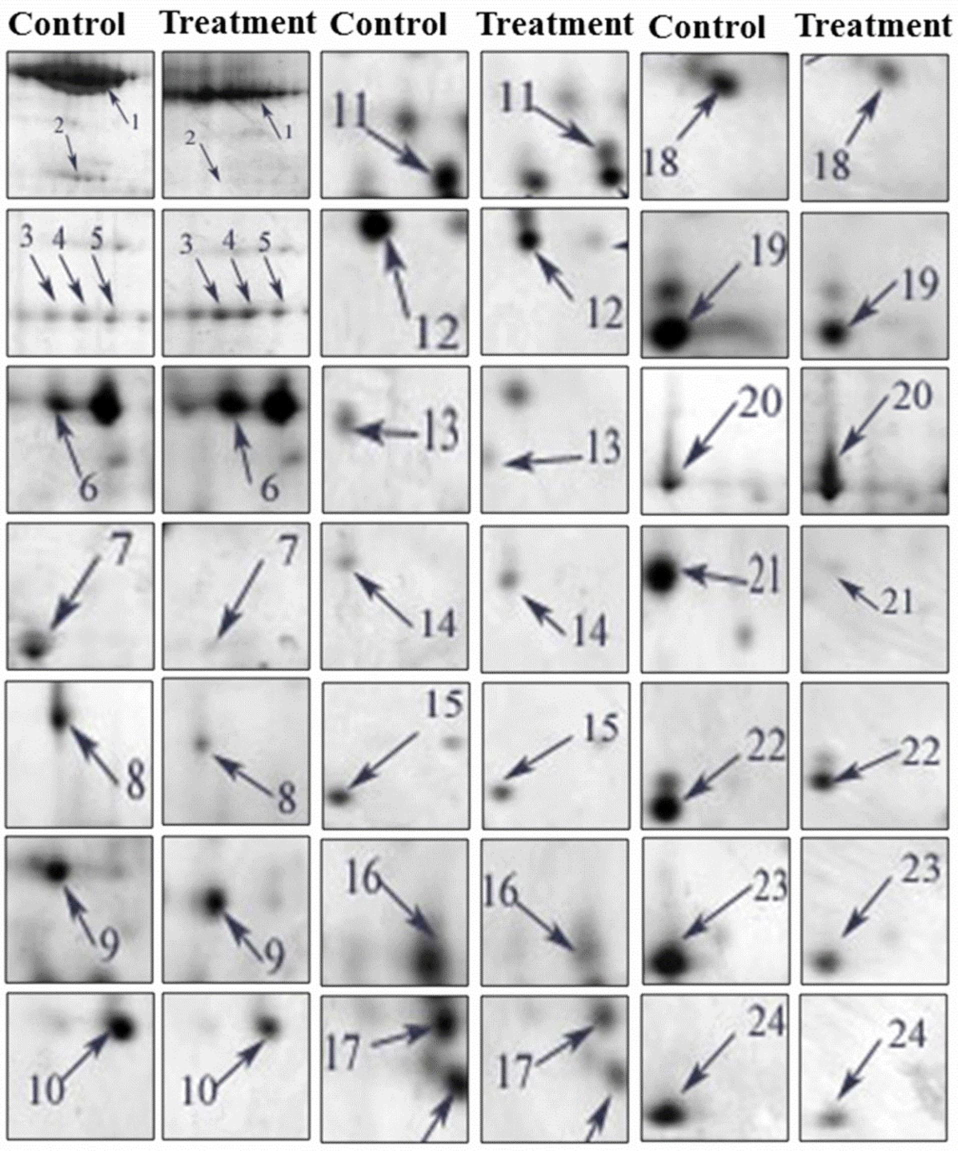


**Figure S2.** Comparative image (control and treatment) of differentially expressed proteins obtained from 2-DE gel in sugarcane variety F134 after *Sporisorium scitamineum* inoculation after 60 days.

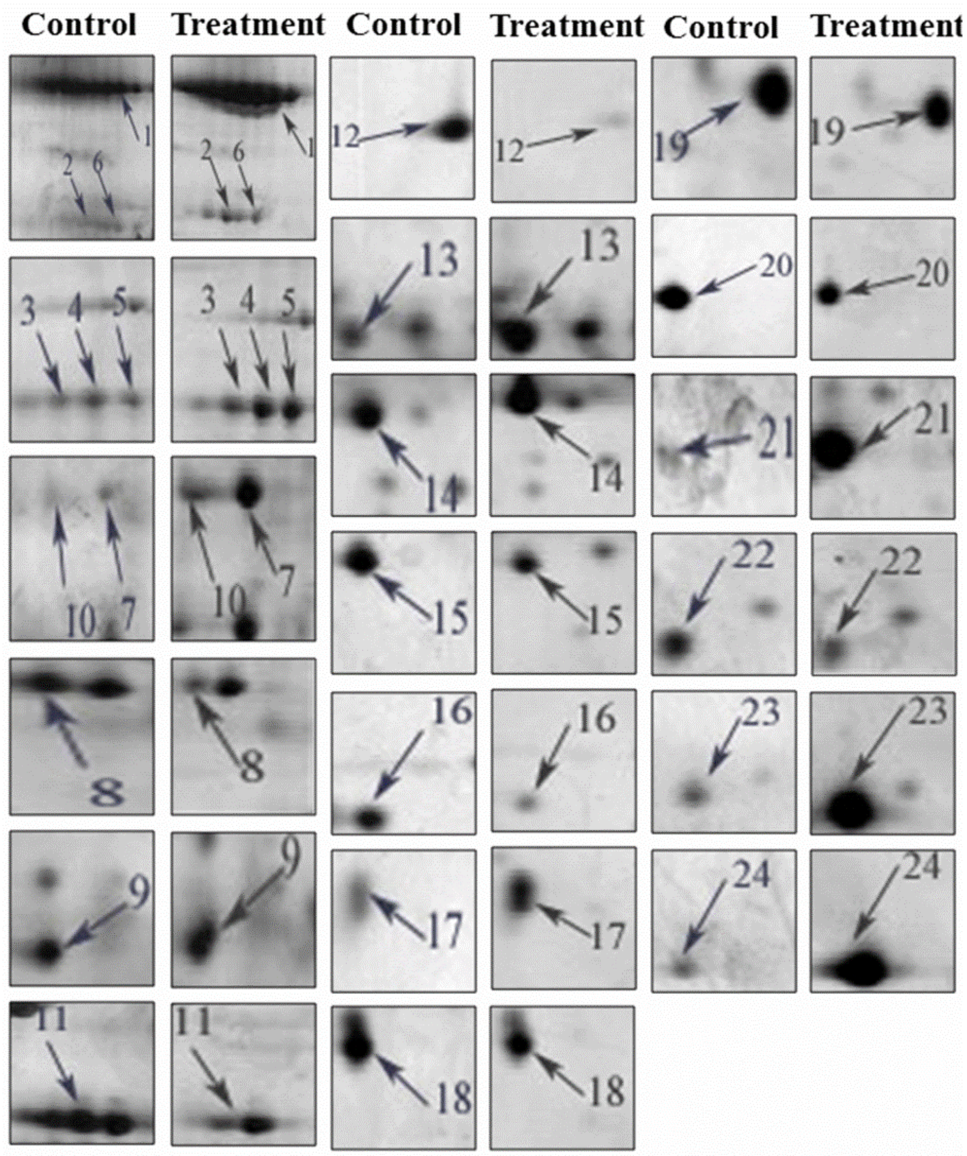


**Figure S3.** Comparative image (control and treatment) of differentially expressed proteins obtained from 2-DE gel in sugarcane variety NCo310 after *Sporisorium scitamineum* inoculation after 60 days.

**Table S1.** Functional characterization and classification of identified protein spots in both sugarcane varieties.

| S. No. | Molecular Function | Sugarcane Varieties | |
|---|---|---|---|
| | | NCo310 | F134 |
| 1. | ATP Binding | 4 | 4 |
| 2. | Kinase Activity | 1 | 1 |
| 3. | Metalion Binding | 1 | 2 |
| 4. | Pyruvate, Phosphate Dikinase Activity | 1 | 1 |
| 5. | Transketolase Activity | 2 | 2 |
| 6. | Calciumion Binding | 1 | 1 |
| 7. | Cobaltion Binding | 1 | 1 |
| 8. | Manganese Ion Binding | 2 | 1 |
| 9. | Phosphoglycerate Kinase Activity | | 1 |
| 10. | Gtpase Activity | 1 | 1 |
| 11. | GTP Binding | 1 | 1 |
| 12. | Translation Elongation Factor Activity | 1 | 1 |
| 13. | Phosphoglycerate Kinase Activity | | 1 |
| 14. | Nucleic Acid Binding | 1 | 1 |
| 15. | Nucleotide Binding | 1 | 1 |
| 16. | Peroxidase Activity | | 1 |
| 17. | DNA Binding | | 1 |
| 18. | Hydrogen Ion Transmembrane Transporter Activity | | 1 |
| 19. | Superoxide Dismutase Activity | | 1 |
| 20. | Monooxygenase Activity | 1 | 1 |
| 21. | Ribulose-Bisphosphate Carboxylase Activity | 1 | 1 |
| 22. | Nutrient Reservoir Activity | 1 | |
| **S. No.** | **Biological Process** | | |
| 1. | Pyruvate Metabolic Process | 1 | 1 |
| 2. | Metabolic Process | 1 | 1 |
| 3. | Reductive Pentose-Phosphate Cycle | 1 | 1 |

| | | | |
|---|---|---|---|
| 4. | Glycolytic Process | | 2 |
| 5. | Response to Glucose | | 1 |
| 6. | Response to Heat | | 1 |
| 7. | Response to Light Stimulus | | 1 |
| 8. | Response to Molecule of Bacterial Origin | | 1 |
| 9. | ATP Synthesis Coupled Proton Transport | | 1 |
| 10. | Defense Response to Bacterium | | 1 |
| 11. | Response to Cytokinin | | 1 |
| 12. | Photorespiration | 1 | 1 |
| 13. | Reductive Pentose-Phosphate Cycle | 1 | 1 |
| 14. | Protein Refolding | 1 | |
| **S. No.** | **Cellular Component** | | |
| 1. | Chloroplast Thylakoid Membrane | 1 | 2 |
| 2. | Intracellular | 1 | 1 |
| 3. | Apoplast | | 1 |
| 4. | Chloroplast Stroma | | 1 |
| 5. | Cytosol | | 1 |
| 6. | Nucleus | | 1 |
| 7. | Plasma Membrane | | 1 |
| 8. | Plasmodesma | | 1 |
| 9. | Vacuolar Membrane | | 1 |
| 10. | Intracellular Ribonucleoprotein Complex | 1 | 1 |
| 11. | Viral Nucleocapsid | 1 | 1 |
| 12. | Chloroplast Envelope | | 1 |
| 13. | Integral Component of Membrane | | 1 |
| 14. | Proton-Transporting ATP Synthase Complex, Coupling Factor F (O) | | 1 |
| 15. | Chloroplast | 1 | 2 |
| 16. | Cytoplasm | 1 | |
| 17. | Extracellular Region | 1 | |
